# Parameter Measurement of Live Animals Based on the Mirror of Multiview Point Cloud

**DOI:** 10.1155/2021/9528787

**Published:** 2021-10-29

**Authors:** Jinsong Zhang, Yue Qin, Li Wang

**Affiliations:** College of Communication and Electronic Engineering, Qiqihar University, Qiqihar 161006, China

## Abstract

Scale and standardization are essential to the prosperity of the breeding industry. During large-scale, standardized breeding, the selective breeding of good livestock breeds hinges on the accurate measurement of body parameters for live animals. However, the complex shooting environment brings several urgent problems, such as the missing of many local data in the point cloud and the difficulty in the automatic acquisition of body data. To solve these problems, this paper proposes a method for parameter measurement of live animals based on the mirror of multiview point cloud. Firstly, the acquisition and stitching principles were given for the multiview point cloud data on body parameters of live animals. Next, the authors presented a way to make up for the data missing areas in the point cloud. Finally, this paper acquires the body mirror data of live animals and scientifically calculates the body parameters. The proposed measurement method was proved effective through experiments.

## 1. Introduction

Precision animal husbandry refers to the scientific breeding and management of live animals by arranging regular daily ration based on information technology. As an important aspect of intelligent agriculture, precision animal husbandry can improve the output benefit of animal husbandry products and ensure product quality and safety [1–4]. Large-scale, standardized breeding can effectively improve the output and profit of pigs, cattle, and sheep. During large-scale, standardized breeding, the selective breeding of good livestock breeds hinges on the accurate measurement of body parameters for live animals [5–12]. The manual measurements with tools like caliper and tap measure are greatly affected by subjective human factors. By contrast, the body measurement of three-dimensional (3D) body parameters, which cover the geometry of live animals, is relatively accurate. The measured data help to assess the health state of livestock, evaluate their body shapes, and identify their behavioral features [13–16].

Focusing on parameter measurement based on 3D point cloud data, Jo et al. [17] relied on the point cloud data of the 3D human body model to construct an objective interpolation function, which can describe the morphological changes of human body (e.g., gender, age, weight, height, and body proportion). Then, the independent elements were weighted reasonably according to the linkage been element changes. In this way, the independent elements were adjusted and updated. After that, the needed human body model was derived from the intermediate human body. Sato [18] proposed a hardware and software system capable of synchronization precise acquisition of point cloud data on live animals. The system consists of an FM810-GI depth camera and its fixation structure, a point cloud data processing module, and a repeater. Rao et al. [19] improved the stereo calibration method of point cloud data on live animals based on the location relationship between the multiple depth cameras used to collect the data. Then, the three-view point cloud data on live animals underwent stitching and duplicate removal by the interactive closest point (ICP) algorithm and k-means clustering (KMC). Finally, a precise 3D point cloud data was established for live animals.

To evaluate the health state of pandas, Turner et al. [20] introduced the skinned multianimal linear (SMAL) model to the 3D model reconstruction of these first class protected animals in China and obtained the base shape and base pose of the 3D panda model based on principal component analysis (PCA) and bone movements. Further, they derived the parameterized description of the shape and pose of the model. Zhang et al. [21] manually extracted animal contours from two-dimensional (2D) images, set up the objective function of Euclidean clustering between SMAL model and contour segmentation maps, and estimated SMAL parameters by minimizing the objective function. Ahsan et al. [22] provided an effective and accurate way to measure the length, width, and depth of pavement cracks. Specifically, watershed segmentation was adopted to segment and mask the background of damaged pavement images, the coordinate system of pavement cracks was converted point by point, a 3D visual model was established on MATLAB for pavement cracks, and the computed results were compared with the measured data. To solve the precision product quality problems induced by manufacturing errors, Chen and Wang [23] proposed a 3D point cloud feature calculation method to compute the geometric and physical parameters of workpieces and combined area changes and centroid deviation into a dense layered part evaluation and adaptive stratification algorithm, which can reconstruct workpiece surfaces and adaptively stratify workpieces.

Some results have been achieved on 3D point cloud and body parameter extraction, as well as weight prediction [24–27]. However, there are often holes in the point cloud, owing to the complex environmental factors, e.g., environmental interference (especially the fences of the breeding base) and low equipment precision. These holes severely impede the postprocessing of the point cloud. In addition, it is very difficult to automatically acquire the body data of live animals [28–31]. To solve these problems, this paper proposes a method for parameter measurement of live animals based on the mirror of multiview point cloud.  Section 2 introduces the acquisition and stitching principles of the multiview point cloud data on body parameters of live animals.  Section 3 presents a way to make up for the data missing areas in the point cloud.  Section 4 acquires the body mirror data of live animals and scientifically calculates the body parameters. The proposed measurement method was proved effective through experiments.

## 2. Data Acquisition and Stitching

During the 3D reconstruction of a specific object, the object must be extracted from the background to ensure the recognition and analysis accuracy. Owing to the complex environment of the breeding base, the point cloud data extracted from live animals contain the background, noises, and outliers. The data need to be preprocessed to remove the background and noises, facilitating further analysis. By visualizing the point cloud data of live animals, it is possible to obtain the left and right point cloud data of the background, including the ground, cameras, and noises. Considering the complex living environment of animals, the point cloud data were extracted from live animals in the following steps: (1) crop the point cloud data in the specified coordinate range, using passthrough filter; (2) remove ground point cloud data through planar template matching; and (3) eliminate outliers with redundant information by statistical filter.

**Figure 1:**
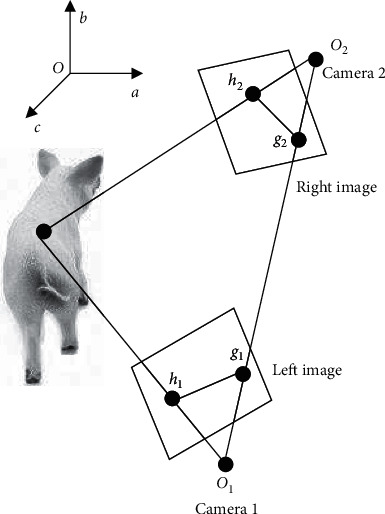
Mirroring principle of multiview point cloud.

Figure 1 shows the mirroring principle of multiview point cloud. It can be inferred that the multiview point cloud data on live animals contain multiple coordinate systems, such as rotation and translation. Point cloud registration is necessary to unify the coordinates of multiple point clouds under different coordinate systems.

Let *G*, *p*, and *U* be the rotation matrix, translation matrix, and perspective transform vector between two depth cameras, respectively, with *U* being a zero vector and *A* = 1 be the proportional factor of the multiview point cloud on live animals. Then, the mapping *F* of point cloud registration can be expressed as follows:

(1) $$\begin{array}{c} F=\left[\begin{array}{cccc} G_{11} & G_{11} & G_{11} & p\\ G_{21} & G_{22} & G_{23} & p\\ G_{31} & G_{32} & G_{33} & p\\ u_{x} & u_{y} & u_{s} & A \end{array}\right]⟶F=\left[\begin{array}{cc} G_{3\times 3} & p_{3\times 1}\\ U_{1\times 3} & A \end{array}\right]. \end{array}$$

To directly stitch point clouds on live animals, it is necessary to determine the location relationship between depth cameras. The parameters can be obtained through the stereo calibration in the binocular visual system. Let *O*_*SJ*_ be the coordinate of any point *O* in the world coordinate system; *G*_1_ and *G*_2_ be the rotation matrices of cameras 1 and 2 relative to the calibration object, respectively; and *p*_1_ and *p*_2_ be the translation matrices of cameras 1 and 2 relative to the calibration object, respectively. Under the world coordinate system, the coordinates of the two cameras can be described by the following:

(2) $$\begin{array}{c} \left\{\begin{array}{c} O_{1}=G_{1}O_{SJ}+p_{1},\\ O_{2}=G_{2}O_{SJ}+p_{2}. \end{array}\right) \end{array}$$

The relationship between *O*_1_ and *O*_2_ can be established as follows:

(3) $$\begin{array}{c} O_{1}=GO_{2}+p. \end{array}$$

Combing formulas (2) and (3),

(4) $$\begin{array}{c} \left\{\begin{array}{c} G=G_{1}\left(G_{2}\right)p,\\ p=p_{1}-Gp_{2}. \end{array}\right) \end{array}$$

According to the affine invariance of four point pairs in 4-point congruent sets (4PCS), the distance ratio *g* can be fixed with three known colinear points *U*, *V*, and *W*:

(5) $$\begin{array}{c} g=\left\|\frac{U-V}{U-W}\right\|. \end{array}$$

Suppose *U* and *W* fall on the same straight line and *V* and *Q* fall on the same straight line. In addition, the two lines intersect at point *H*. Then, distance ratios *g*_1_ and *g*_2_ can be calculated by the following:

(6) $$\begin{array}{c} g_{1}=\left\|\frac{U-H}{U-W}\right\|,\\ g_{2}=\left\|\frac{V-H}{V-Q}\right\|. \end{array}$$

During affine transform, the distance ratios *g*_1_ and *g*_2_ determined by the four coplanar points of the source point cloud and the corresponding four points in the target point cloud are constant, i.e., completely the same. If there exists any point pair *s*_1_ and *s*_2_ in *S* whose lines intersect at points *h*_1_ and *h*_2_, which are the same within a certain error range, then *s*_1_ and *s*_2_ are the coplanar points corresponding to the given base in the world coordinate system. The intersections *h*_1_ and *h*_2_ can be calculated by the following:

(7) $$\begin{array}{c} h_{1}=s_{1}+g_{1}\left(s_{2}-s_{1}\right),\\ h_{2}=s_{1}+g_{2}\left(s_{2}-s_{1}\right). \end{array}$$

If the point cloud data on live animals are stitched directly using the results of stereo calibration, the registration accuracy needs to be guaranteed through iterations by the precision matching algorithm *ICP*. Suppose the point set under the world coordinate system and the target point set is denoted as *O* = {*o*_*i*_*|o*_*i*_∈ℝ^3^, *i* = 1, 2,…, *m*} and *S* = {*s*_*j*_|*s*_*j*_∈ℝ^3^, *j* = 1,2,…, *n*}, respectively. Under the premise of minimizing the error function error(*G*, *p*) between the two point sets in formula (8), the least squares method can be adopted to iteratively perform the optimal coordinate transform and calculate the rotation matrix and translation matrix until the preset error threshold or maximum number of iterations is reached:

(8) $$\begin{array}{c} \text{error}\left(G,p\right)=\frac{1}{m}\sum_{i=1}^{m}{\left\|s_{i}-\left(Go_{i}+p\right)\right\|}^{2}. \end{array}$$

## 3. Repairing Missing Areas

To make up for the large nonclosed missing areas in the point cloud of live animals, this paper proposes the cubic B-spline curve fitting method based on the projections of point cloud slices.

The slicing of the point cloud on live animals was carried out along the *a*-axis. The first step is to determine the minimum distance *ε*_min_ between the point cloud center and other points and the maximum *a*_max_ and minimum *a*_min_ of the center along the *a*-axis. Next, point cloud splices were sampled from *a*_min_ in the positive direction of *a*-axis, with an interval of *ε*_min_. The sampling number *M*_*S*_ can be calculated by the following:

(9) $$\begin{array}{c} M_{S}=\left\lceil \frac{a_{\max}-a_{\min}}{\varepsilon_{\min}}\right\rceil , \end{array}$$

where the square brackets stand for rounding operation. The sampling interval of the *i*-th point cloud slice *O*_*i*_ can be described as [*a*_min_ + (*i* − 1) *ε*_min_, *a*_min_ + *iε*_min_]. Then, the maximum *b*_*i* − max_ and minimum *b*_*i* − min_ of *O*_*i*_ along *b*-axis were determined, and the point cloud was sliced into *M*_*i*_ parts with an interval of *ε*_min_:

(10) $$\begin{array}{c} M_{i}=\left\lceil \frac{b_{i-\max}-a_{i-\min}}{\varepsilon_{\min}}\right\rceil . \end{array}$$

The curve fitting effect is greatly affected by the number of new points appearing through the expansion of the interval of point cloud slices. Therefore, this paper selects the center *O*_*il*_ of the *l*-th interval [*b*_*i* − min_ + (*l* − 1) *ε*_min_, *b*_*i* − min_ + *l ε*_min_] of *O*_*i*_ long *b*-axis as the representative point of that interval. Suppose the interval contains *N*_*l*_ points, with *O*_*ilk*_ being the *k*-th point. Then, we have

(11) $$\begin{array}{c} O_{il}=\frac{1}{N_{l}}\sum_{j=1}^{N_{l}}O_{ilk}. \end{array}$$

The processed *O*_*i*_ was projected onto plane boc. The projection point was then fitted. When restoring the fitted point cloud *O*_*i*_^*∗*^ to the space, the points in interval *O*_*i*_^*∗*^ along *a*-axis should be configured uniformly:

(12) $$\begin{array}{c} a_{i}^{*}=a_{\min}+\left(p-\frac{1}{2}\right)\cdot \varepsilon_{\min}. \end{array}$$

Suppose the slice plane or space of the point cloud on live animals contains *u*+*v*+1 vertices. Then, *O*_*i*_ has a *v*-dimensional parametric curve segment:

(13) $$\begin{array}{c} O_{jv}\left(p\right)=\sum_{j=0}^{u}O_{j+l}R_{j,v}\left(p\right). \end{array}$$

The *v*-dimensional B-spline curve can be derived from the *v*-dimensional B-spline curve segment *O*_*jv*_(*p*) above. The base function *R*_*iv*_(*p*) of the curve can be calculated by the following:

(14) $$\begin{array}{c} R_{iv}\left(p\right)=\frac{1}{v!}\sum_{t=0}^{v-t}{\left(-1\right)}^{t}W_{v+1}^{t}{\left(p+v-i-t\right)}^{v}. \end{array}$$

The *v*-dimensional B-spline curve can be defined by *v* − 1 adjacent vertices. Then, the cubic B-spline curve can be expressed as follows:

(15) $$\begin{array}{c} O_{j3}\left(p\right)=\sum_{i=0}^{3}O_{i+j}R_{i,3}\left(p\right). \end{array}$$

The corresponding base function can be expressed as follows:

(16) $$\begin{array}{c} \left\{\begin{array}{c} R_{0,3}\left(p\right)=\frac{1}{6}\left(-p^{3}+3p^{2}-3p+1\right),\\ R_{1,3}\left(p\right)=\frac{1}{6}\left(3p^{3}-6p^{2}+4\right),\\ R_{2,3}\left(p\right)=\frac{1}{6}\left(-3p^{3}+3p^{2}+3p+1\right),\\ R_{3,3}\left(p\right)=\frac{1}{6}p^{3}. \end{array}\right) \end{array}$$

The *j*-th segment of the cubic B-spline curve can be described by the following:

(17) $$\begin{array}{c} O_{j3}\left(p\right)=\frac{1}{6}\left[\begin{array}{cccc} 1 & p & p^{2} & p^{3} \end{array}\right]\left[\begin{array}{cccc} 1 & 4 & 1 & 0\\ -3 & 0 & 3 & 0\\ 3 & -6 & 3 & 0\\ -1 & 3 & -3 & 1 \end{array}\right]\left[\begin{array}{c} O_{j}\\ O_{j+1}\\ O_{j+2}\\ O_{j+3} \end{array}\right]. \end{array}$$

**Figure 2:**
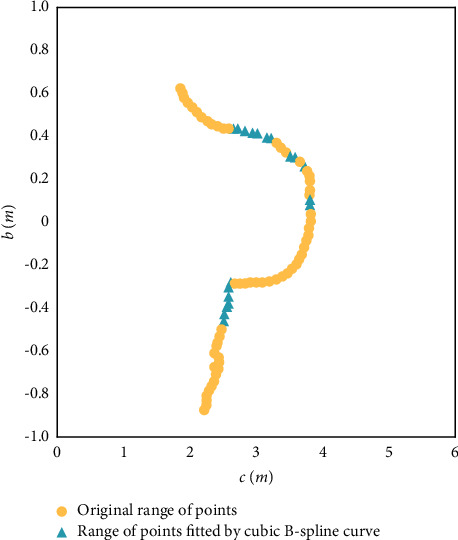
Projection and fitting of point cloud slices on forelimbs of live animals.

Figure 2 shows the projection and fitting of point cloud slices on the forelimbs of a cow. After the projection and fitting, the distance between adjacent points averaged at 6.842 mm, the standard deviation was 1.514 mm, and the approximate error was 0.426 mm. The number of points increased by 67.2% to 270. The fitted range of points was close to the original range of points.

## 4. Mirror Data Acquisition and Parameter Calculation

### 4.1. Key Point Positioning and Mirror Data Acquisition

**Figure 3:**
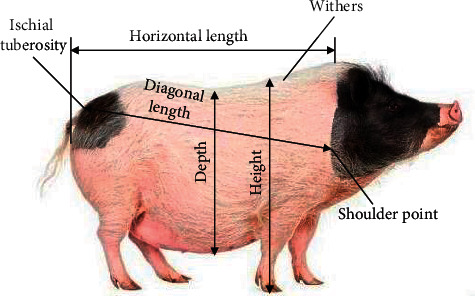
Side view of a live animal.

Figure 3 presents the side view of a live animal. To obtain the exact values of the body parameters, it is necessary to locate the key points of the body of the live animal. Cattle, pigs, and sheep share the same key points, including the point of maximum abdominal width, the shoulder point and its transition points, the point of ischial tuberosity, and the point of withers.

During the preprocessing, the ground plane equation was defined for each frame of the point cloud. The side view and top view correspond to planes aob and aoc, respectively. Specifically, the point of maximum abdominal width *P*_1_ is the point furthest away from the line connecting the left and right end points in the fitted point range. The transition point *P*_2_ of the shoulder point *P*_4_ characterizes the point at which the slope of the cloud segment *P*_1_–*P*_4_ turns from positive to negative. Along the positive direction of axis *a*, the number *M*_*EK*_ of centers in the cloud segment *P*_1_–*P*_4_ was calculated with *P*_1_ (*a*_1_, *c*_1_) as the starting point. Then, the angle *ω*_*i*_ between axis *a* and the line connecting *P*_1_ with each point in *O*_*i*_ (*a*_*o*_, *c*_*o*_) (*i* = 1,2,…,*M*_*EK*_) can be calculated by the following:

(18) $$\begin{array}{c} \omega_{i}=\arctan\left|\frac{c_{o_{i}}-c_{1}}{a_{o_{i}}-a_{1}}\right|. \end{array}$$

The nearby transition point *P*_3_ at which the slope also turns from positive to negative was identified in a similar manner as point *P*_2_. Along the positive direction of axis *a*, the number MFK of centers in the cloud segment *P*_1_–*P*_4_ was calculated with *P*_2_ (*a*_2_, *c*_2_) as the starting point. Then, the angle *ω*_*j*_ between axis *a* and each point in *O*_*i*_ (*a*_*o*_, *c*_*o*_) (*i* = 1,2,…, MFK) with *P*_1_ (*a*_1_, *c*_1_) as the starting point and satisfying *c*_*oj*_ ≥ *c*_1_ can be obtained by the following:

(19) $$\begin{array}{c} \left\{\begin{array}{c} a_{1}=a_{c_{\min}},c_{1}=\frac{c_{\min}+c_{\max}}{2},\\ \omega_{j}=\arctan\left|\frac{c_{o_{i}}-c_{1}}{a_{o_{i}}-a_{1}}\right|,\\ c_{p_{i}}\geq c_{1}. \end{array}\right) \end{array}$$

After obtaining *P*_2_ and *P*_3_, the shoulder point of the live animal *P*_4_ was determined as the farthest point in the point cloud segment *P*_2_-*P*_3_ from the line connecting *P*_2_ and *P*_3_. Then, the point of ischial tuberosity *P*_5_ could be obtained as the center of the K nearest points to the point of minimum *a*. After that, the point of withers *P*_6_ could be solved by computing the center coordinates of all the tallest points in the 2 slice point clouds extended to the left and right of the axis *a* coordinates of the midpoint of *P*_2_ and *P*_4_. Finally, the upper point *P*_*U*_ and lower point *P*_*D*_ of the depth could be solved by computing the center coordinates of all the tallest points and all the lowest points in the 2 slice point clouds extended to the left and right of the axis *a* coordinates of point *P*_1_, respectively.

To get an accurate plane of symmetry for the body of the live animal, the normal vector *γ*_*op*_ of the ground supporting the animal and the horizontal direction vector *ξ*_*p*_ of the animal were aligned with the positive directions of axes *a* and *b*, respectively, to normalize the poses. Then, the normal vector *ϕ*_*p*_ of the plane of symmetry is the product between *γ*_*op*_ and *ξ*_*p*_:

(20) $$\begin{array}{c} \varphi_{p}=\gamma_{op}\times \xi_{p}. \end{array}$$

The above analysis shows that the tail point of the live animal is the extreme point in the negative direction of axis *a*, whose coordinates are (*a*_0_, *b*_0_, *c*_0_). From (*a*_0_, *b*_0_, *c*_0_) and *ϕ*_*p*_, the planar equation of the live animal can be determined as *c* = *c*_0_. The mirror data on one side of the plane of asymmetry could be obtained by setting up the homogeneous coordinates of the point *O*_*t*1_ = {(*a*, *b*, *c*)|*c* > *c*_0_} on one side of the plane:

(21) $$\begin{array}{c} \left[\begin{array}{cccc} a^{\prime } & b^{\prime } & c^{\prime } & 1 \end{array}\right]=\left[\begin{array}{cccc} a & b & c & 1 \end{array}\right]\left[\begin{array}{cccc} 1 & 1 & 1 & 0\\ 0 & 1 & 0 & 0\\ 0 & 0 & -1 & 0\\ 0 & 0 & 2c_{0} & 1 \end{array}\right]. \end{array}$$

After obtaining the symmetric data *O*_*t*2_ = {(*a'*, *b'*, *b'*|*a'*, *b'*, *c* ∈ *O*_*t*2_} of the live animal, it is assumed that *O*_*t*_ = *O*_*t*1_ + *O*_*t*2_, where *O*_*t*_ is the mirror of the point cloud on the complete animal in the 3D space.

### 4.2. Calculation of Body Parameters

The Euclidean distance from *P*_4_(*a*_4_, *b*_4_, *c*_4_) to *P*_5_(*a*_5_, *b*_5_, *c*_5_) was defined as the diagonal length of the Euclidean distance:

(22) $$\begin{array}{c} \zeta_{O}=\sqrt{{\left(a_{4}-a_{5}\right)}^{2}+{\left(b_{4}-b_{5}\right)}^{2}+{\left(c_{4}-c_{5}\right)}^{2}}. \end{array}$$

The horizontal distance from *P*_4_ (*a*_4_, *b*_4_, *c*_4_) to the vertical line of *P*_5_ (*a*_5_, *b*_5_, *c*_5_) was defined as the horizontal distance:

(23) $$\begin{array}{c} \zeta_{S}=\left|a_{4}-a_{5}\right|. \end{array}$$

The shoulder width was defined as twice the distance from *P*_4_(*a*_4_, *b*_4_, *c*_4_) to the plane of symmetry *c*=*μ*_0_^*∗*^+*μ*_1_*a*:

(24) $$\begin{array}{c} \psi_{J}=\frac{\left|\mu_{1}a_{4}-c_{4}+\mu_{0}^{*}\right|}{\sqrt{\mu_{1}^{2}+1}}. \end{array}$$

The abdominal width was defined as twice the distance from *P*_1_ (*a*_1_, *b*_1_, *c*_1_) to *c*=*μ*_0_^*∗*^+*μ*_1_*a*:

(25) $$\begin{array}{c} \psi_{F}=\frac{\left|\mu_{1}a_{5}-c_{5}+\mu_{0}^{*}\right|}{\sqrt{\mu_{1}^{2}+1}}. \end{array}$$

The height was defined as the distance from *P*_6_ (*a*_6_, *b*_6_, *c*_6_) to the ground *τ*_*a*_*a* *+* *τ*_*b*_*b* *+* *τ*_*c*_*c* *+* *υ* = 0:

(26) $$\begin{array}{c} H_{A}=\frac{\left|\tau_{a}a_{6}+\tau_{b}b_{6}+\tau_{c}c_{6}+\upsilon \right|}{\sqrt{\tau_{a}^{2}+\tau_{b}^{2}+\tau_{c}^{2}}}. \end{array}$$

The depth was defined by the vertical heights of *P*_*U*_ (*a*^*P*^_*U*_, *b*^*P*^_*U*_, *c*^*P*^_*U*_) and *P*_*D*_ (*a*^*P*^_*D*_, *b*^*P*^_*D*_, *c*^*P*^_*D*_):

(27) $$\begin{array}{c} T_{u,\upsilon }=\left|c_{P_{U}}-c_{P_{D}}\right|. \end{array}$$

## 5. Experiments and Result Analysis

**Figure 4:**
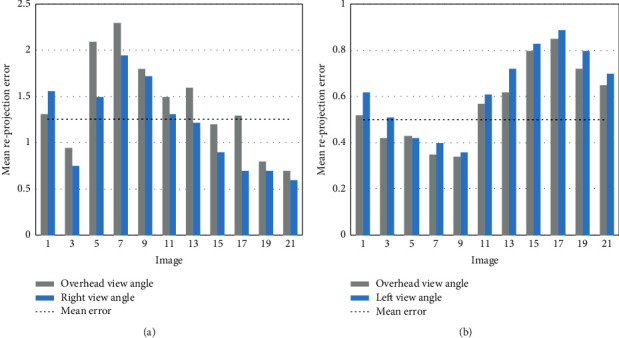
Mean reprojection errors of overhead, left, and right depth cameras: (a) overhead and right cameras; (b) overhead and left cameras.

During the acquisition of point cloud data from live animals, it is difficult to shoot an image and complete 3D calibration using normal calibration targets. Thus, this paper performs 3D calibration with large and small infrared calibration targets. Depending on the deployment of depth cameras, the overhead camera was calibrated separately with the left infrared lens of the left camera and the right infrared lens of the right camera. The calibration errors are recorded in Figures 4(a) and 4(b). It can be inferred that the mean reprojection error between overhead camera and the left infrared lens of right camera was 1.31 pixels, and that between overhead camera and the right infrared lens of left camera was 0.87 pixels. Both results meet the precision requirements.

**Figure 5:**
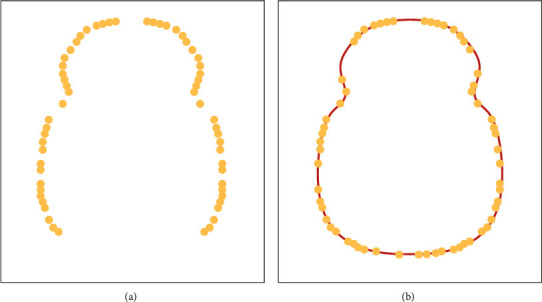
Curves fitted from chest circumference measuring points: (a) passthrough filter; (b) our method.

The axis *a* coordinates of chest circumference measuring points on a live pig were recorded on interactive measuring software of point cloud data. The fitting parameters were set as follows: the order of the curve, 4; the number of iterations, 50; and the number of control points, 100.  Figure 5(a) shows the point cloud within 0.005 before and after the coordinates obtained by passthrough filter.  Figure 5(b) shows the curve obtained by our cubic B-spline curve fitting method, which is marked in red. The curve circumference could be estimated from the length of the approximate polygon composed of the curve control points.

**Figure 6:**
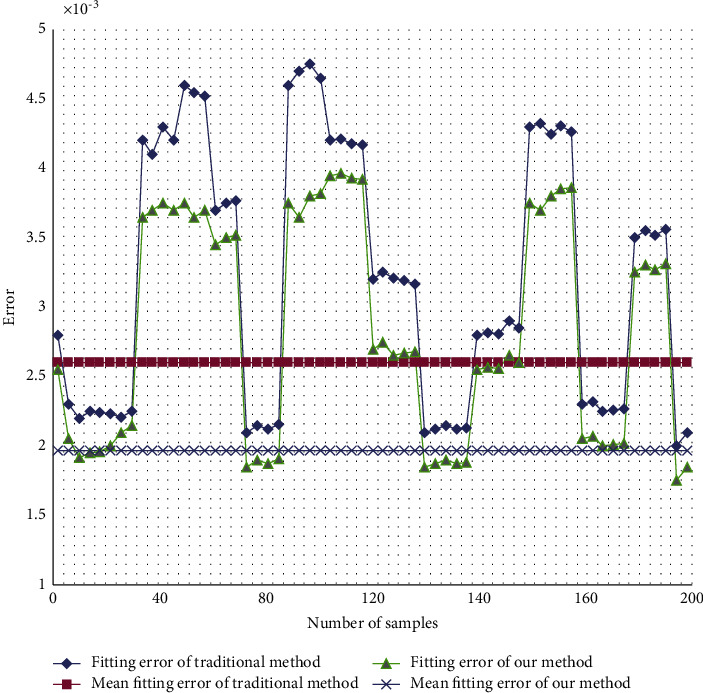
Errors of different methods in missing area repairing.

Our point cloud repairing method was applied to the missing areas in the 240 frames of point clouds on 50 pigs. These areas went missing due to the occlusions of railings. The fitting errors of the traditional method and our method are displayed in Figure 6. The mean, maximum, and minimum fitting errors of traditional cubic B-spline curve were 2.524 mm, 4.452 mm, and 2.346 mm, respectively; those of our method, i.e., cubic B-spline curve fitting based on projection of point cloud slices, were 1.924 mm, 3.754 mm, and 1.859 mm, respectively. This further confirms that the curve fitted by our method is closer to the original point cloud.

To verify its effectiveness, the proposed algorithm was compared with two other models through experiments. The processing results of different models are listed in Table 1. Our algorithm achieved relatively good results on segmenting live animals in point cloud data: the recall was as high as 82.7% and the accuracy as 88.9%. The recall and accuracy of region growth + threshold judgement were 80.4% and 82.7%, respectively. The recall and accuracy of watershed segmentation were merely 55.1% and 80.4%, respectively. The comparison shows that our algorithm boasts a good precision and high recall and accuracy. As for the two contrastive models, region growth + threshold judgement outperformed watershed segmentation in both recall and accuracy.

**Figure 7:**
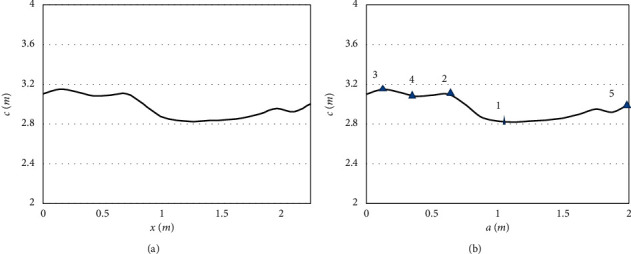
(a) Point range projection. (b) Key point identification.

The body appearance of live animals can be well displayed by the side view captured by depth cameras and characterized by the projection of point range on plane aoc. In general, the local curves of the projected point range are not smooth enough despite being relatively simple. The local point range could be fitted ideally, using a cubic polynomial in one variable with 2 extremes. The fitting effect is shown in Figure 7(a). The key points of live animal body are presented in Figure 7(b), including the point of maximum abdominal width *P*_1_, the shoulder point *P*_4_ and its transition points *P*_2_ and *P*_3_, and the point of ischial tuberosity *P*_5_. The positions of the point cloud on the live animal projected to the 3D space could be derived from the projected position of each of these points.

**Figure 8:**
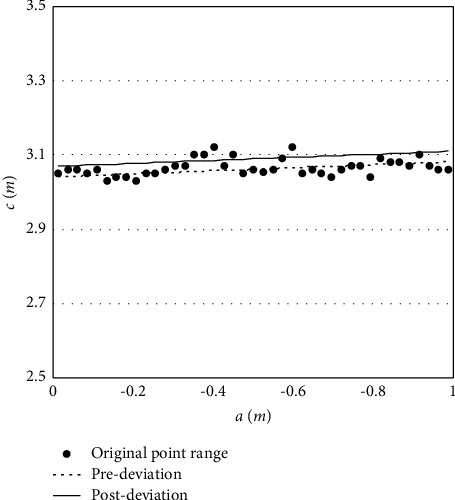
Fitted line of symmetry.

The point cloud data obtained from one side only cover half of the body of the live animal. This calls for the restoration of the mirror of the point cloud. After determining the symmetric longitudinal section along the line connecting the center of the head and the center of the tail of the live animal, the shoulder width is equivalent to twice the distance from the point of maximum shoulder width to the plane of symmetry. Similarly, the abdominal width is equivalent to twice the distance from the point of maximum abdominal width to the plane of symmetry. The tallest points on the point cloud slices between the following points were merged into a point range: point of maximum abdominal width *P*_1_, shoulder point *P*_4_ and its transition points *P*_2_ and *P*_3_, and point of ischial tuberosity *P*_5_. Then, the outliers were removed from the point range, and the center coordinates were solved. The resulting new point range was fitted by a linear equation. Then, the straight line was translated to the mean distance from every point in the range to the center coordinates, producing the line of symmetry of the live animal. Then, it is possible to derive the mirror data of the point cloud of that animal.  Figure 8 shows the fitted line of symmetry.

The body parameters were extracted and measured from the 240 frames of point clouds on 50 pigs. Table 1 presents the measured results.  Table 2 compares the point cloud measurements with manual measurements.

As shown in Table 3, the MAE of height measurement was minimized at 0.0032. The MAEs of other parameters were within 0.0270. Specifically, diagonal length and horizontal length had relatively large MAEs (0.0262 and 0.0232), 35% greater than the MAEs of other parameters. The MRE of height was also minimized at 0.9127%. The MRE of horizontal length was 5.0327, above the MREs of all the other five parameters. Regardless of MAE or MRE, the measurement errors of horizontal and diagonal lengths were relatively large, while those of height and depth were small. The main reason is that the slight changes of body position of the live animals during the measurement affects the accuracy of key point identification. Besides, the subjectiveness of manual measurement also influences the determination of key points. Of course, the errors were relatively small. The above results show that our measurement method for body parameters is accurate enough for application.

## 6. Conclusions

This paper develops a parameter measurement method for live animals based on the mirror of multiview point cloud. After being acquired from the target animal, the point cloud data from multiple views were preprocessed and stitched, followed by the eliminating of redundant background points. Next, the features of the point cloud data were analyzed, and a 3D point cloud data model was established for live animals. After that, the authors explained how to repair the missing part of the point cloud data, acquired the mirror data on animal body, and scientifically computed the body parameters. Experimental results confirm the scientific nature of the calibrating overhead camera separately with the left infrared lens of the right camera and the right infrared lens of the left camera. In addition, the chest circumference measuring points were fitted into a curve, and the errors of different methods were compared for repairing missing areas in the point cloud. The relevant results demonstrate the effectiveness of our fitting algorithm. Further, the line of asymmetry of a live animal was fitted, which proves the feasibility and effectiveness of our point cloud acquisition method. Finally, the measuring errors of body parameters were presented, suggesting the high accuracy of our body parameter measuring method for live animals.

## Figures and Tables

**Table 1:** Processing results of different models.

Model	Live animals	Number of samples	Number of effectively segmented samples	Number of oversegmented samples	Number of undersegmented samples	Recall	Accuracy
Watershed segmentation	Pigs	154	87	15	34	55.1	77.1
	Cattle	227	103	22	29	54.3	80.4

Region growth + threshold judgement	Pigs	154	99	14	31	67.8	81.4
	Cattle	227	134	21	22	80.4	82.7

Our algorithm	Pigs	154	139	14	30	74.2	88.9
	Cattle	227	188	20	21	82.7	83.7

**Table 2:** Measured results of body parameters.

Serial number	Diagonal length	Horizontal length	Shoulder width	Abdominal width	Height	Depth
1	1.6335	1.6312	0.3125	0.7802	1.4125	0.7936
2	1.8203	1.7536	0.5327	0.8231	1.4257	0.8752
3	1.5721	1.5264	0.5346	0.7523	1.4329	0.8534
4	1.3592	1.6317	0.6371	0.6375	1.3546	0.7580
5	1.6317	1.7322	0.3511	0.7438	1.4728	0.8375
6	1.7692	1.7605	0.3670	0.6782	1.4159	0.8351
7	1.7326	1.5632	0.5234	0.9357	1.3072	0.8439
8	1.6545	1.4725	0.5602	0.7521	1.3821	0.8217
9	1.5217	1.6241	0.5437	0.6325	1.5057	0.7536
10	1.6039	1.5492	0.6539	0.7253	1.3723	0.8419

**Table 3:** Measurement errors.

	Diagonal length	Horizontal length	Shoulder width	Abdominal width	Height	Depth
Mean absolute error (MAE)	0.0262	0.0232	0.0193	0.0185	0.0032	0.0096
Mean relative error (MRE)	4.9235	5.0327	3.9752	2.4371	0.9127	1.1358

## Data Availability

The data used to support the findings of this study are available from the corresponding author upon request.
